# Knowledge and Attitudes Toward Telemedicine Among Family Medicine Residents in Riyadh, Saudi Arabia: An Observational Cross-Sectional Study

**DOI:** 10.7759/cureus.65655

**Published:** 2024-07-29

**Authors:** Razan K Alhadlaq, Amal A Afrah, Maya T Mohiden, Samaher Z Alsaad

**Affiliations:** 1 Family Medicine, King Saud Medical City, Riyadh, SAU; 2 Family Medicine, Riyadh Third Health Cluster, Riyadh, SAU

**Keywords:** saudi arabia, attitudes, knowledge, family medicine physicians, telemedicine

## Abstract

Background

Telemedicine is an emerging concept that involves the use of electronic information and communications technologies to provide and support healthcare. This study aimed to assess the knowledge and attitudes of family medicine residents toward telemedicine in Riyadh, Saudi Arabia.

Methodology

This was a cross-sectional observational study using a self-administered questionnaire distributed among family medicine residents in Saudi Arabia. The collected data included sociodemographic features, residents' knowledge, and attitudes toward telemedicine. Data were collected in Excel and analyzed using SPSS software version 29. A P-value of less than 0.05 was considered statistically significant.

Results

The study included 279 family medicine residents with a median age of 27 years. The majority of participants (n=191, 68.5%) had a good level of knowledge regarding telemedicine, which was significantly associated with younger age (p-value = 0.012). Additionally, there was an overall good attitude toward telemedicine attributes, significantly associated with older age and female gender (p-values <0.05).

Conclusion

This study highlights that family medicine residents in Riyadh, Saudi Arabia, generally possess good knowledge and positive attitudes towards telemedicine. Key findings indicate that younger residents are more knowledgeable about telemedicine, while older residents and female participants show more favorable attitudes toward its attributes. However, further training and education on the ease of use of telemedicine applications are required.

## Introduction

The WHO defines telemedicine as 'the delivery of health care services, where distance is a critical factor, by all health care professionals using information and communication technologies for the exchange of valid information for diagnosis, treatment, and prevention of disease and injuries, research and evaluation, and for the continuing education of health care providers, all in the interests of advancing the health of individuals and their communities' [1].

Telemedicine plays a crucial role in providing healthcare services to different parts of the world, facilitating access to all populations, especially for those who live in areas where it is challenging to access usual or face-to-face health services [2]. There is a demand for national healthcare systems to deliver fast, easily accessible, high-quality healthcare using economical methods. Telehealth utilizes technological audio and visual tools to provide real-time communication between the patient and the healthcare professional during the delivery of remote health services.

Telehealth aims to provide clinical suggestions, consultations, treatment, education, and administrative services while also monitoring patients' health [3]. To enhance the quality and usability of healthcare services for patients and healthcare professionals, particularly in rural and distant locations, the Ministry of Health (MoH) in Saudi Arabia recently launched an e-health plan that incorporates telemedicine [4].

Family medicine is defined as 'continuing, comprehensive medical care of the patient in the context of the family and community. Continuing whole-patient care incorporates prevention, diagnosis, and treatment of undifferentiated illness; acute and chronic care; recognition of family and social needs; long-term support; and epidemiologic and environmental awareness' [5].

A primary healthcare system is the backbone and first line of defense for any healthcare system, and family medicine is one of its most crucial pillars. Primary care plays a major role in the healthcare system as the initial point of contact for patients seeking care and as the gatekeeper. Interest in investigating the possibilities of telemedicine in addressing many of the difficulties affecting primary care in the United States and around the world has been driven by growing concerns about sustainability and the predicted shortages of primary care physicians [6].

Historically, telemedicine emerged when a Dutch physicist first created an electrocardiograph and used it to record the electrical cardiac signals of patients in a hospital 11 km away [7]. It is used for various functions, from real-time teleconsultation between doctors and patients to performing major innovations through robotic surgeries [8].

A study conducted in 2016 in Minnesota collected data from 1,285 ambulatory clinics exploring the factors affecting the adoption of telemedicine. It showed that only 55% adopted telemedicine, and 26% of those clinics adopted real-time tele-consultation services [9]. Locally, in Saudi Arabia, the MoH introduced several forms of telemedicine as an alternative to face-to-face consultations in clinical settings. MoH released more than 19 smartphone applications to provide health services to smartphone users, constituting 96% of the population [10]. Another key initiative to improve healthcare access for those without an internet connection was the establishment of 937, a free, confidential telephone service that provides medical and administrative healthcare services at any time [10].

Many studies conducted worldwide have shown telemedicine's importance in supporting primary healthcare. A randomized controlled trial in Norway showed that patients with secure messaging access to the primary clinic made fewer office visits, saving patients' time and effort [11]. Additionally, it showed advantages for patients with chronic diseases. The impact of remote monitoring on patients with type 2 diabetes showed that participation in telehealth was associated with considerable HbA1c reductions [12]. It also has a positive outcome in obstetrics for female patients with gestational diabetes mellitus (GDM) compared to in-person visits [13].

Observing physicians' knowledge and attitudes toward telemedicine is essential to ensure the proper usage of technology and also to satisfy patient outcomes. Especially family physicians, as they are the first line of defense for the community. Locally, in Taif, physicians view that there were limitations related to patient confidence in revealing privacy-related issues and how the management would be affected without a complete physical examination and laboratory data [14]. Additionally, data from a study conducted in the Riyadh region assessing knowledge and attitude toward telemedicine showed high levels of perceptions of participants; on the contrary, almost half of the participants had significantly low levels of knowledge [4].

The majority of healthcare workers in a study conducted in India were partly in agreement that telemedicine should be used in all hospitals with internet facilities as it will facilitate more accessible access to healthcare for patients in rural areas, save travel time and costs, as well as reduce patient time and expenses [15]. A cross-sectional study conducted in Germany among family physician trainees addressing the attitude towards telemedicine in 2016 showed that around two-thirds of the participants thought that only a fraction of telemedicine is currently implemented, and one of the significant barriers was data safety concerns [16, 17].

Therefore, this study aimed to assess the knowledge and attitudes of family medicine residents toward telemedicine in Riyadh city.

## Materials and methods

Study design and setting

This was a cross-sectional observational study conducted using a self-administered questionnaire that was disseminated among family medicine residents in Riyadh, Saudi Arabia.

Study duration

The original study was conducted from March to May 2024, after obtaining ethical approval in March 2024. However, the study was planned, piloted, and sent for approval from January to December 2023.

Eligibility criteria

All family medicine residents at any level (Residency Level 1 (R1), Residency Level 2 (R2), and Residency Level 3 (R3)) in Riyadh were included in this study. However, residents in specialties other than family medicine and those working outside Riyadh were excluded for the following reasons: (1) data collection was conducted specifically in Riyadh for direct access to residents and to ensure a higher response rate; (2) only family medicine residents were included to provide a specialty-centered viewpoint for focused improvements in residency training; (3) to maintain a national focus on improving telemedicine, especially in primary healthcare services.

Sampling technique

A convenience sampling technique was employed to recruit participants for this study. The Riyadh region hosts multiple family medicine residency programs, each with criteria for resident distribution. This sampling technique was chosen to account for the possibility of a poor response rate.

Sample size calculation

The sample size was calculated using an online calculator (https://calculator.net). With a confidence interval of 95% and a 5% margin of error, given a population size of family medicine residents in Riyadh estimated at 440, the required sample size was calculated to be 206.

Data collection tool

The questionnaire was primarily based on a Mayo Clinic questionnaire, while the modified version was obtained from a similar study conducted in Ethiopia [18]. It consists of 36 items divided into three main sections. The first section collects demographic information, including gender, age, and level of residency (R1, R2, R3). The second section includes ten questions assessing the knowledge of telemedicine. The third and final section, which assesses attitudes toward telemedicine, contains 23 questions divided into five main categories: relative advantages of telemedicine (seven items), compatibility of telemedicine (four items), complexity of telemedicine deployment (five items), trialability of telemedicine application’s ease of use (four items), and observability of telemedicine (three items). The questionnaire was validated using face and content validity methods, and its reliability was calculated using Cronbach’s alpha coefficient (α = 0.73).

Statistical analysis

Knowledge of telemedicine was assessed using a binary (yes/no) response, where 'yes' was scored as 1 and 'no' as 0. A mean score of 5 or below indicated poor knowledge, while a score above 6 reflected good knowledge. Attitudes toward telemedicine across five categories were scored on a 5-point scale ranging from 1 (strongly disagree) to 5 (strongly agree), except for complexity attribute questions, which were scored reversely. Attitudes were analyzed by averaging scores for each attribute, with a mean score below 2.5 indicating a poor attitude, 2.6-3.0 indicating a moderate attitude, and above 3.0 indicating a good attitude.

Statistical analysis was conducted using SPSS version 29.0. Descriptive statistics, including frequencies, percentages, and medians with IQR, were used, along with inferential tests such as Pearson Chi-square/Fisher's Exact test, Mann-Whitney, and Kruskal-Wallis tests to explore associations between demographic characteristics and knowledge/attitude scores. A significance level of less than 0.05 was considered.

Ethical consideration 

This study was approved by the Research Ethics Committee of King Saud Medical City via reference number H1R1-12-Feb24-01, dated 11-03-2024. Informed consent was clarified at the beginning of the questionnaire, stating that personal data would be kept anonymous, and the investigators ensured the confidentiality of the data collected.

## Results

The study involved the assessment of 279 family medicine residents in Riyadh, Saudi Arabia. The median age of participants was 27 years, with an interquartile range spanning from 26 to 28 years. The gender distribution was almost equal, with 49.1% (n=137) male participants. The residency levels varied, with approximately 38% (n=106) at R3 level (Table 1).

**Table 1:** Sociodemographic data of the family medicine residents (n=279). The data were presented in frequency (n) and percentage (%), while age was presented in the median, mean, and IQR. R1: Residency level 1; R2: Residency level 2; R3: Residency level 3.

Variable	Frequency (Percentage) n (%)
Age (years) Median (IQR) = 27 (26-28), Mean (SD)= 27.54 (1.79)	
Gender	
Male	137 (49.1)
Female	142 (50.9)
Level of Residency	
R1	100 (35.8)
R2	73 (26.2)
R3	106 (38.0)

It was found that the majority demonstrated a good level of knowledge (n=191, 68.5%), while 31.5% (n=88) exhibited poor knowledge.

**Figure 1:**
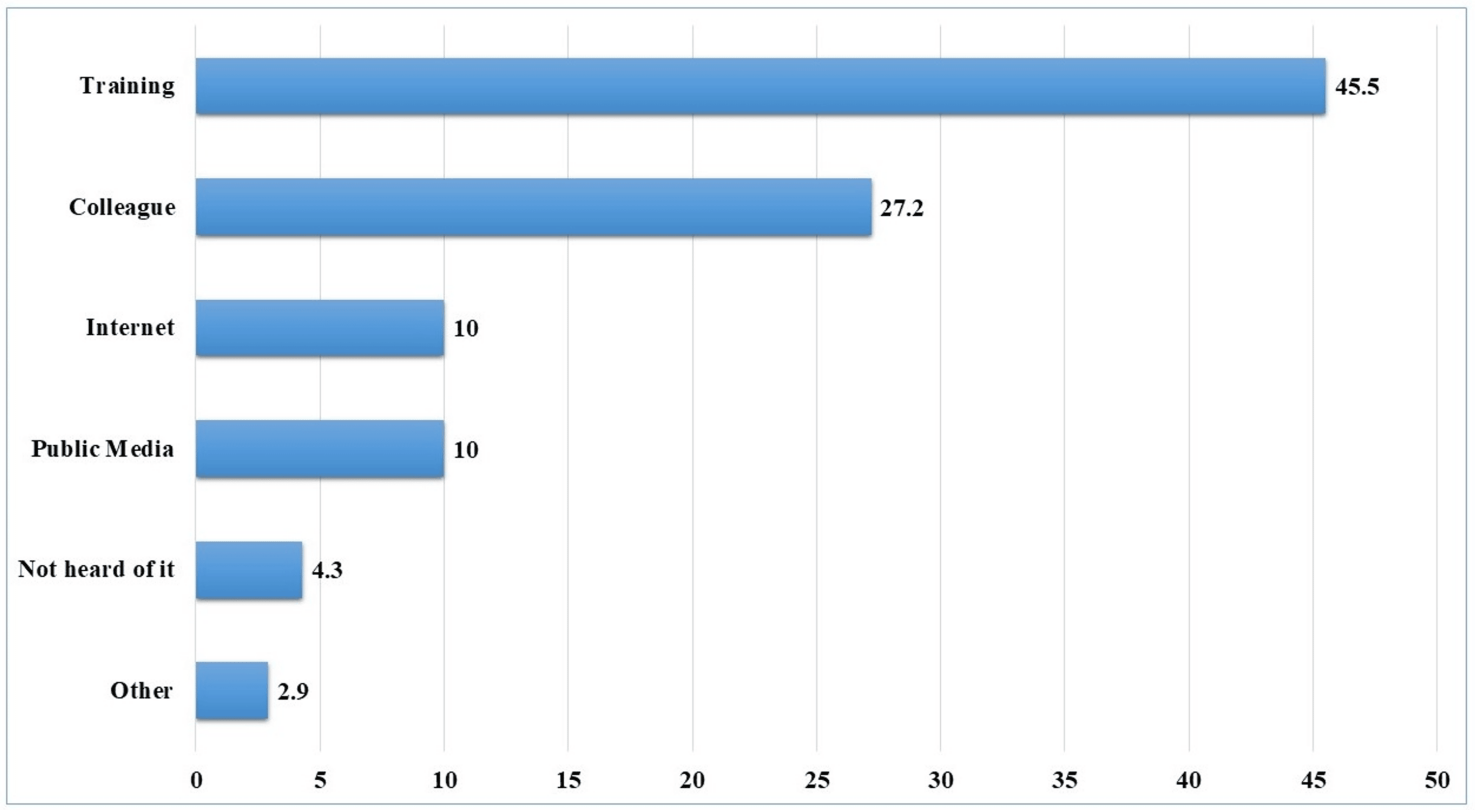
Sources of information regarding telemedicine among the family medicine residents. The data were presented as percentage (%).

Most participants had prior knowledge of telemedicine (n=251, 90%). This knowledge was acquired through various sources, including training courses (n=127, 45.5%), colleagues (n=76, 27.2%), and the internet (n=28, 10%) (Figure 1).

A significant number of participants recognized the benefits of telemedicine, such as saving clinicians’ time (n=237, 84.9%), reducing unnecessary transportation costs (n=229, 82.1%), and decreasing the need for medical staff (n=209, 74.9%). Additionally, more than half of the participants were familiar with telemedicine tools such as telesurgery, teleconsultation, and teleconferencing (n=149, 53.4%). Interestingly, only 38% (n=106) were knowledgeable about telemedicine infrastructure (Table 2).

**Table 2:** Knowledge of family medicine residents regarding telemedicine (n=279). The data were presented in frequency (n) and percentage (%).

Knowledge of Telemedicine		Frequency (Percentage) n (%)
Have you ever heard about telemedicine?	Yes	251 (90.0)
	No	28 (10.0)
I know the benefits of telemedicine in saving clinicians’ time.	Yes	237 (84.9)
	No	42 (15.1)
I know the benefits of telemedicine in reducing unnecessary transportation costs.	Yes	229 (82.1)
	No	50 (17.9)
I know the effect of telemedicine on reducing medical staff's needs.	Yes	209 (74.9)
	No	70 (25.1)
I know the effect of telemedicine on healthcare quality.	Yes	199 (71.3)
	No	80 (28.7)
I know telemedicine technology	Yes	189 (67.7)
	No	90 (32.3)
I know telemedicine tools like telesurgery, teleconsultation, teleconferencing, etc.	Yes	149 (53.4)
	No	130 (46.6)
Have you ever seen a telemedicine system?	Yes	147 (52.7)
	No	132 (47.3)
I know about telemedicine infrastructure.	Yes	106 (38.0)
	No	173 (62.0)

The analysis revealed a significant association between age and knowledge level (p-value = 0.012), indicating that younger residents, with a median age of 27 years, exhibited better knowledge of telemedicine compared to their older counterparts, with a median age of 28 years (Table 3).

**Table 3:** Association of knowledge score of family medicine residents and sociodemographic data. The data were presented in frequency (n) and percentage (%). M: P-value generated through Mann-Whitney test, C: P-value generated through Pearson Chi-square test.

Sociodemographic characteristics	Knowledge level		P-value
	Good n (%)	Poor n (%)	
Total	191 (68.5)	88 (31.5)	
Age (years)			
Median (IQR)	27 (26-28)	28 (27-29)	0.012^M^
Mean (SD)	27.34 (1.63)	27.98 (2.06)	
Gender			
Male	98 (69.0)	44 (31.0)	0.898^C^
Female	93 (67.9)	44 (32.1)	
Residency Level			
R1	66 (66.0)	34 (34.0)	0.358^C^
R2	55 (75.3)	18 (24.7)	
R3	70 (66.0)	36 (34.0)	
The overall knowledge score (median + IQR): R1: 6.5 (3-9), R2: 7 (5-8), R3: 6 (4-8.25)			0.728

**Figure 2:**
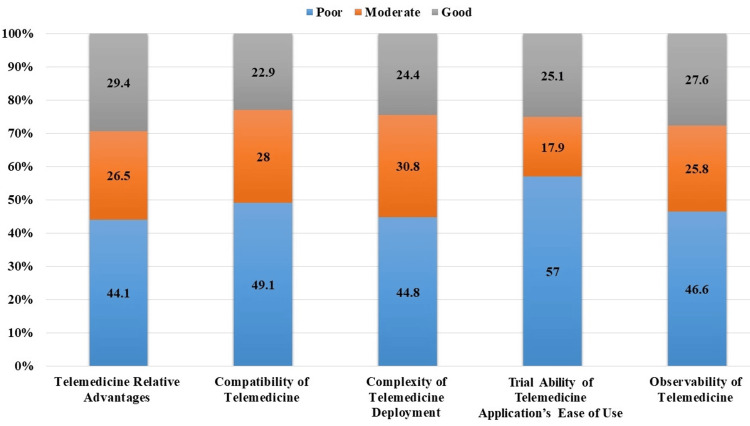
Intensity of family medicine residents' attitudes towards each telemedicine attribute. The data were presented as percentage (%).

Overall, the questions related to telemedicine relative advantages reflected the highest level of positive attitudes among participants, with 29.4% (n=82) expressing favorable views. Conversely, questions concerning the trialability of telemedicine application’s ease of use received the lowest level of positive attitudes, with 57% (n=159) expressing unfavorable views (Figure 2).

Significant relationships were found between participants' age and their attitudes towards both telemedicine relative advantages (p = 0.005) and complexity of telemedicine deployment (p = 0.025). Older participants tended to exhibit moderate attitudes towards these attributes, with a mean age of 28.03 years for telemedicine relative advantages questions and 27.91 years for complexity of telemedicine deployment questions, compared to other age groups (Table 4).

**Table 4:** Association of attitude level of family medicine residents and their age. The data were presented in median and mean. *P-value was generated through the Kruskal-Wallis test. Poor attitude: a mean score ≤ 2.5, moderate score: 2.6-3.0, good score: ≥3.1.

Telemedicine Attribute	Attitude	Age (Median)	P-value*
Telemedicine Relative Advantages (7 item)	Poor	27 (26-28)	0.005
	Moderate	28 (27-29)	
	Good	27 (26-28)	
Compatibility of Telemedicine (4 items)	Poor	27 (26-28)	0.904
	Moderate	28 (26-28.25)	
	Good	27 (27-29)	
Complexity of Telemedicine Deployment (5 items)	Poor	27 (27-28.50)	0.025
	Moderate	27 (27-29)	
	Good	27 (26-28)	
Trial Ability of Telemedicine Application’s Ease of Use (4 items)	Poor	27 (26-28)	0.972
	Moderate	27.50 (25.25-29.75)	
	Good	27 (26-29)	
Observability of Telemedicine (3 items)	Poor	27 (26-28)	0.536
	Moderate	27 (26-29)	
	Good	27 (26-28)	

Female practitioners exhibited a higher proportion of positive and good attitudes towards telemedicine relative advantages (n=52, 38%, p-value= 0.007), compatibility of telemedicine (n=34, 24.8%, p-value= 0.038), complexity of telemedicine deployment (n=46, 33.6%, p-value=0.002), and observability of telemedicine (n=45, 32.8%, p-value=0.016), compared to males (Table 5).

**Table 5:** Association of attitude level of family medicine residents and their gender. The data were presented in frequency (n) and percentage (%). *P-value was generated through Pearson Chi-square test.

Telemedicine Attribute	Attitude	Gender n (%)		P-value*
		Female	Male	
Telemedicine Relative Advantages (7 items)	Poor	51 (37.2)	72 (50.7)	0.007
	Moderate	34 (24.8)	40 (28.2)	
	Good	52 (38.0)	30 (21.1)	
Compatibility of Telemedicine (4 items)	Poor	57 (41.6)	80 (56.3)	0.038
	Moderate	46 (33.6)	32 (22.5)	
	Good	34 (24.8)	30 (21.2)	
Complexity of Telemedicine Deployment (5 items)	Poor	53 (38.7)	72 (50.7)	0.002
	Moderate	38 (27.7)	48 (33.8)	
	Good	46 (33.6)	22 (15.5)	
Trial Ability of Telemedicine Application’s Ease of Use (4 items)	Poor	73 (53.3)	86 (60.6)	0.415
	Moderate	28 (20.4)	22 (15.5)	
	Good	36 (26.3)	34 (23.9)	
Observability of Telemedicine (3 items)	Poor	52 (38.0)	78 (54.9)	0.016
	Moderate	40 (29.2)	32 (22.5)	
	Good	45 (32.8)	32 (22.5)	

Family medicine residents within the second residency level (R2) exhibited more positive and good attitudes towards the trialability of telemedicine application’s ease of use (n=26, 35.7%, p-value=0.001), while those in the third residency level (R3) showed a higher proportion of positive attitudes towards telemedicine relative advantages (n=36, 34%, p-value= 0.024), compatibility of telemedicine (n=28, 26.5%, p-value= 0.018), and observability of telemedicine (n=36, 34%, p-value=0.001) compared to their counterparts in other residency levels (Table 6).

**Table 6:** Association of attitude level of participants and their level of residency. The data were presented in frequency (n) and percentage (%). *P-value was generated through the Pearson Chi-square test K: P-value was generated through the Kruskal-Wallis test. R1: Residency Level 1; R2: Residency Level 2; R3: Residency Level 3.

Telemedicine Attribute	Attitude	Level of Residency n (%)			P-value*
		R1	R2	R3	
Telemedicine Relative Advantages (7 items)	Poor	36 (36.0)	43 (58.9)	44 (41.5)	0.024
	Moderate	34 (34.0)	14 (19.2)	26 (24.5)	
	Good	30 (30.0)	16 (21.9)	36 (34.0)	
Compatibility of Telemedicine (4 items)	Poor	42 (42.0)	41 (56.2)	54 (50.9)	0.018
	Moderate	40 (40.0)	14 (19.2)	24 (22.6)	
	Good	18 (18.0)	18 (24.6)	28 (26.5)	
Complexity of Telemedicine Deployment (5 items)	Poor	48 (48.0)	25 (34.2)	52 (49.1)	0.208
	Moderate	28 (28.0)	30 (41.1)	28 (26.4)	
	Good	24 (24.0)	18 (24.7)	26 (24.5)	
Trial Ability of Telemedicine Application’s Ease of Use (4 items)	Poor	50 (50.0)	45 (61.6)	64 (60.4)	0.001
	Moderate	30 (30.0)	2 (2.7)	18 (17.0)	
	Good	20 (20.0)	26 (35.7)	24 (22.6)	
Observability of Telemedicine (3 items)	Poor	40 (40.0)	36 (49.3)	54 (50.9)	0.001
	Moderate	40 (40.0)	16 (21.9)	16 (15.1)	
	Good	20 (20.0)	21 (28.8)	36 (34.0)	
Overall Attitude Score (median+ IQR)		13.7 (11-15.4)	13.3 (10.2-16.6)	12.0 (10-16.1)	0.797^K^

## Discussion

Information technology (IT) aids in improving medical services, for example, telemedicine and eHealth. Telemedicine is a general term used to describe telehealth, electronic medical records (EMRs), eHealth, and other health IT components [19]. Telemedicine and eHealth involve the use of electronic information and advanced telecommunication technologies to deliver remote clinical healthcare, manage patients and their medical records, and implement targeted professional health-related training, public health, and health management [19, 20]. According to the World Health Organization, eHealth is the combined application of information and communication technology (ICT) for health [20]. Proper application of telemedicine can help improve patients' access to cost-effective and high-quality healthcare services [21]. In this study, the knowledge and attitude of family medicine residents in Riyadh towards telemedicine were assessed.

The current study results showed that the majority of the family medicine practitioners (n=191, 68.5%) demonstrated a good level of knowledge about telemedicine, and 90% (n=251) had prior knowledge, mostly from training (n=45.5%). These findings are similar to those in a previous cross-sectional study by Barnawi NA et al., which showed that the majority of healthcare providers demonstrated a good level of knowledge [22]. Moreover, another cross-sectional study by Alajwari HA et al. reported that the majority of Saudi citizens were familiar with the term ‘telemedicine’ and believed that telemedicine could reduce transportation costs [23]. Another study by Ahmed TJ et al. reported that most young doctors and nurses had a good level of knowledge about telemedicine, and nearly half of the participants thought that telemedicine is more useful for chronic medical patients [24]. These are promising findings for the implementation of telemedicine programs in Saudi Arabia, as there are several situations in which telemedicine has great importance, such as during emergency conditions like the COVID-19 pandemic [25-28].

Most of the participants recognized the importance of telemedicine in saving clinicians' time (n=237, 84.9%), reducing unnecessary transportation costs (n=229, 82.1%), decreasing the need for medical staff (n=209, 74.9%), enhancing healthcare quality (n=199, 71.3%), and being familiar with its technology (n=189, 67.7%). More than half of the participants were familiar with telemedicine tools and systems (n=149, 53.4%). Conversely, in assessing clinicians' knowledge about telemedicine in a study conducted in Iran by Ayatollahi H et al., researchers reported that the majority of clinicians had a poor level of knowledge, with a moderate level of perception regarding the advantages of using telemedicine technology [29]. The variations among studies' findings might be due to the extent of improvements in telemedicine training and practicability now compared to before, also the aforementioned study included different categories of clinicians and nurses.

The current study results showed that most participants expressed a relatively positive attitude toward the advantages of telemedicine (n=156, 55.9%) and the complexity of telemedicine deployment (n=154, 55.2%). However, several responders showed a poor attitude toward the trialability of telemedicine application’s ease of use (n=159, 57%). A similar study conducted in Ethiopia reported the same results regarding individuals’ attitudes toward telemedicine advantages. However, a dominance of a good attitude toward its compatibility and a poor attitude toward the complexity and observability of telemedicine were observed [18].

A significant association between age and knowledge level was found, showing that younger residents, with a median age of 27 years, exhibited better knowledge of telemedicine compared to their older counterparts. Also, a significant relationship was found between participants' age and their attitudes towards both telemedicine relative advantages and the complexity of telemedicine deployment, with participants' mean age of 28.03 years showing a moderate attitude toward telemedicine relative advantages, and participants of 27.91 years showing a moderate attitude toward the complexity of telemedicine deployment. This differs from what was previously reported by Barnawi NA et al., who showed no association between primary healthcare providers' knowledge and attitude about telemedicine and their ages [22]. The difference is that Barnawi NA et al. included PHC providers with different professional backgrounds, so different ages represent different career points for different health providers.

According to the current study results, female practitioners showed a higher proportion of positive/good attitudes towards telemedicine relative advantages, compatibility of telemedicine, complexity of telemedicine deployment, and observability of telemedicine. This differs from what was previously reported by Elhadi M et al., who found no significant association between attitude and gender; however, their study showed a significant difference between included male and female age groups [29].

According to the current study results, practitioners within the second residency level (R2) exhibited more positive/good attitudes toward the trialability of telemedicine application’s ease of use, while those in the third residency level (R3) showed a higher proportion of positive attitudes towards telemedicine relative advantages, compatibility, and observability of telemedicine. This indicated an increase in positive attitudes toward telemedicine with an increase in years of practice. However, a previous study found no significant association between years of experience and telemedicine attitude among doctors in Saudi Arabia [22].

The current study encountered several limitations. Despite a sample size of 279 residents in Riyadh, the study offers insights into family medicine residents' attitudes towards telemedicine. It suggests a need for a larger and more diverse sample and deeper exploration of factors influencing attitudes and knowledge. Incorporating qualitative data could provide richer insights. While the study's cross-sectional design captures current attitudes, it lacks insights into actual telemedicine practices among residents. Despite its limitations, the study informs local policies and training programs in Riyadh, highlighting demographic groups that may benefit from targeted interventions.

Future studies should analyze barriers to telemedicine adoption, such as technological challenges, privacy concerns, and resistance to change, to inform targeted interventions. Longitudinal studies tracking changes in knowledge and attitudes over time as telemedicine technology and policies evolve would provide valuable insights into the long-term impact of telemedicine education.

## Conclusions

This study highlights that family medicine residents in Riyadh, Saudi Arabia, generally possess good knowledge and positive attitudes towards telemedicine. Key findings indicate that younger residents are more knowledgeable about telemedicine, while older residents and female participants show more favorable attitudes toward its attributes. Despite the study’s limitations, including its localized focus and cross-sectional design, the insights gained are valuable for informing local policies, training programs, and resource allocation. Future research should further explore barriers to telemedicine adoption and the effectiveness of targeted interventions and training programs to enhance telemedicine knowledge and attitudes among medical residents.

## Appendices

Appendix 1

Family medicine residents’ knowledge and attitudes towards telemedicine questionnaire.

**Table 7:** Sociodemographic information.

Item		
1	Gender	Female
		Male
2	Age	
3	Level of Residency	R1
		R2
		R3

**Table 8:** The knowledge of family medicine residents toward telemedicine.

Item	Question	Yes	No
1	Have you ever heard about telemedicine		
2	If yes, what was your source of information? Training. Colleague Public Media Internet Other		
3	Have you ever seen telemedicine system?		
4	I know telemedicine technology		
5	I know telemedicine tools like tele surgery, teleconsultation, teleconferencing and so on		
6	I know the effect of telemedicine on healthcare quality		
7	I know the effect of telemedicine on reducing medical staffs needed		
8	know about telemedicine infrastructure		
9	I know the benefits of telemedicine on reducing the unnecessary transportation cost		
10	I know the benefits of telemedicine in saving clinicians time		

**Table 9:** The attitude of family medicine residents toward telemedicine.

Item	I believe telemedicine may	Strongly disagree	Disagree	Neutral	Agree	Strongly agree
	Relative Advantage					
11	Reduce medical errors					
12	Facilitate diagnosis and treatment					
13	Increase communication among health care providers					
14	telemedicine can reduce the number of visits to healthcare centers					
15	Enables me accomplish my task more quickly					
16	Improve clinical decisions					
17	Provide more comprehensive healthcare services					
	Compatibility					
18	In my opinion, telemedicine is compatible with all aspects of my work					
19	telemedicine is completely compatible with my current situation					
20	I think telemedicine fits well with the way I like to work					
21	Using telemedicine fits well into my work style					
	Complexity					
22	I believe using telemedicine requires a lots of mental effort*					
23	Learning to operate telemedicine is hard for me*					
24	I think telemedicine Increase staff work load*					
25	I think telemedicine Create new responsibilities for staff*					
